# Behaviour change techniques in home-based cardiac rehabilitation: a systematic review

**DOI:** 10.3399/bjgp16X686617

**Published:** 2016-08-02

**Authors:** Neil Heron, Frank Kee, Michael Donnelly, Christopher Cardwell, Mark A Tully, Margaret E Cupples

**Affiliations:** NIHR clinical academic fellow in GP/sport and exercise medicine, Centre for Public Health, School of Medicine, Dentistry and Biomedical Science, Queen’s University, Belfast; and The UK Clinical Research Collaboration, Centre of Excellence for Public Health (Northern Ireland), Institute of Clinical Science B, Royal Victoria Hospital, Belfast, UK; Centre for Public Health, School of Medicine, Dentistry and Biomedical Science, Queen’s University, Belfast; and The UK Clinical Research Collaboration, Centre of Excellence for Public Health (Northern Ireland), Institute of Clinical Science B, Royal Victoria Hospital, Belfast, UK; Centre for Public Health, School of Medicine, Dentistry and Biomedical Science, Queen’s University, Belfast; and The UK Clinical Research Collaboration, Centre of Excellence for Public Health (Northern Ireland), Institute of Clinical Science B, Royal Victoria Hospital, Belfast, UK; Centre for Public Health, School of Medicine, Dentistry and Biomedical Science, Queen’s University, Belfast; and The UK Clinical Research Collaboration, Centre of Excellence for Public Health (Northern Ireland), Institute of Clinical Science B, Royal Victoria Hospital, Belfast, UK; Centre for Public Health, School of Medicine, Dentistry and Biomedical Science, Queen’s University, Belfast; and The UK Clinical Research Collaboration, Centre of Excellence for Public Health (Northern Ireland), Institute of Clinical Science B, Royal Victoria Hospital, Belfast, UK; Centre for Public Health, School of Medicine, Dentistry and Biomedical Science, Queen’s University, Belfast; and The UK Clinical Research Collaboration, Centre of Excellence for Public Health (Northern Ireland), Institute of Clinical Science B, Royal Victoria Hospital, Belfast, UK.

**Keywords:** coronary artery bypass grafting, GPs, heart failure, myocardial infarction, review, systematic, secondary prevention

## Abstract

**Background:**

Cardiac rehabilitation (CR) programmes offering secondary prevention for cardiovascular disease (CVD) advise healthy lifestyle behaviours, with the behaviour change techniques (BCTs) of goals and planning, feedback and monitoring, and social support recommended. More information is needed about BCT use in home-based CR to support these programmes in practice.

**Aim:**

To identify and describe the use of BCTs in home-based CR programmes.

**Design and setting:**

Randomised controlled trials of home-based CR between 2005 and 2015 were identified by searching MEDLINE^®^, Embase, PsycINFO, Web of Science, and Cochrane Database.

**Method:**

Reviewers independently screened titles and abstracts for eligibility. Relevant data, including BCTs, were extracted from included studies. A meta-analysis studied risk factor change in home-based and comparator programmes.

**Results:**

From 2448 studies identified, 11 of good methodological quality (10 on post-myocardial infarction, one on heart failure, 1907 patients) were included. These reported the use of 20 different BCTs. Social support (unspecified) was used in all studies and goal setting (behaviour) in 10. Of the 11 studies, 10 reported effectiveness in reducing CVD risk factors, but one study showed no improvement compared to usual care. This study differed from effective programmes in that it didn’t include BCTs that had instructions on how to perform the behaviour and monitoring, or a credible source.

**Conclusion:**

Social support and goal setting were frequently used BCTs in home-based CR programmes, with the BCTs related to monitoring, instruction on how to perform the behaviour, and credible source being included in effective programmes. Further robust trials are needed to determine the relative value of different BCTs within CR programmes.

## INTRODUCTION

Cardiovascular diseases (CVD) are leading causes of death, with survivors often being left with considerable morbidity and disability.^1^ Cardiac rehabilitation (CR) is an effective form of secondary prevention for patients with CVD.^2^^,^^3^ It is a complex health service intervention with behaviour change techniques (BCTs) integral within its design, aiming to assist patients to improve adherence to health-related behaviours to deliver changes in different modifiable vascular risk factors. Medical Research Council (MRC) guidelines^4^^,^^5^ advise the application of behaviour change theory within complex health service interventions, and its use within the evaluation of interventions, to allow greater understanding of exactly how behaviour change is occurring.^6^

The National Institute of Health and Care Excellence (NICE) published guidance in 2014 on individual-level behaviour change interventions for promoting change in modifiable cardiovascular risk factors.^7^ These guidelines indicate that the three behaviour change areas most positively associated with promoting change in modifiable vascular risk factors are goals and planning, feedback and monitoring, and social support. These areas correspond to Michie’s BCT taxonomy.^8^ This is a taxonomy of 93 hierarchically clustered techniques used to facilitate behaviour change within interventions.

There is strong evidence via systematic reviews and meta-analysis^2^ to support the use of CR programmes, including home-based approaches, in a patient population with CVD, particularly for those who have experienced a myocardial infarction (MI). Yet, despite the evidence for this positive treatment option for vascular secondary prevention, there is not a clear understanding of how this complex health service intervention influences behaviour change related to modifiable cardiovascular risk factors. No previous reviews have been identified that have examined programme components in the context of Michie’s BCT taxonomy.^8^ The aim of this review is therefore to identify the BCTs that have been used in home-based CR programmes, and to describe the frequency of their use in programmes that were effective in reducing CVD risk factors.

Although rehabilitation and secondary prevention programmes following a cardiovascular event are well-evidenced,^3^ there has been little focus on the use of specific BCTs, particularly within programmes in the setting of the patient’s home. An understanding of which BCTs are being utilised is important to allow more accurate replication in implementation of the intervention, both within clinical practice and in research. Understanding which BCTs are being used allows an exploration of causal pathways, thus allowing intervention refinement by either reducing content to that which is working, or improving aspects that are not working. This systematic review aims to help identify the particular BCTs that are associated with improvements in specific modifiable cardiovascular risk factors, and to contribute to an evidence base on which home-based rehabilitation interventions can be further developed and refined for use with CVD patients.

**How this fits in**

Cardiac rehabilitation (CR) is effective in promoting secondary cardiovascular prevention, and includes advice about healthy lifestyle behaviours, but little is known about the use of behaviour change techniques (BCTs) recommended in supporting behaviour change in home-based programmes. This meta-analysis of studies published between 2005 and 2015 confirms that home-based CR is as effective as hospital- and centre-based CR in reducing cardiovascular disease (CVD) risk factors. The BCTs involving social support, goal setting, monitoring, instructions on how to perform the behaviour, and credible source were found in studies of programmes reporting changes in CVD risk factors. Awareness of these BCTs should help primary care practitioners to support the delivery of home-based CR, but further research should examine the relative values of different BCTs within these programmes.

## METHOD

The protocol for this review has been previously published.^9^ The Behaviour Change Taxonomy v1^8^ was used to identify the specific BCTs used within included studies. The lead authors have attended a training workshop run by the developers of the taxonomy, and one of the authors is a recognised ‘expert coder’.

This systematic review is reported in line with the Preferred Reporting Items for Systematic Reviews and Meta-analyses (PRISMA) guidance.^10^^,^^11^ Criteria for considering studies for this review have included human randomised and quasi-randomised controlled trials of home-based CR programmes initiated following a cardiovascular event, for example, post-MI or following a heart failure exacerbation. The review focused on adults, males and females, aged ≥18 years. Any home-based CR programme — a rehabilitation programme being defined by previous authors^2^ and delivered within the home environment — initiated following a cardiovascular event were eligible for inclusion. The authors excluded studies that purely reviewed, for example, an exercise or training programme for the patient. The analysis included trials with a control group, and trials with multiple intervention arms (comparing different types of rehabilitation interventions). The review did not include population or community-wide interventions.

### Outcome measures

The particular BCTs included in home-based CR programmes used for cardiovascular secondary prevention, classified using Michie’s Taxonomy, were identified. In addition, recording the frequency of use of BCTs in programmes that reported reductions in CVD risk factors was undertaken. CVD risk factor outcomes in effective studies were used for a meta-analysis of the differences in effect between home-based and comparator programmes.

### Search methods for identification of studies

Detailed search strategies were developed for each electronic database searched, including Ovid MEDLINE^®^ 1946 to June 2015, Ovid Embase 1974 to June 2015, EBSCO Cumulative Index to Nursing and Allied Health Literature (CINAHL) plus 1937 to June 2015, Cochrane Database and Ovid PsycINFO 1806 to June 2015. The searches were based on the strategy developed for Medical Literature Analysis and Retrieval System Online (MEDLINE) and revised appropriately (information available from authors).

The titles and abstracts of publications obtained by the search strategy were independently screened by two authors. Articles that did not meet the inclusion criteria were removed. All remaining publications were retrieved for further assessment. Two review authors selected the trials eligible for inclusion in the review with, if necessary, a third review author resolving disagreements. A record was kept of all articles excluded at this stage and the reason for their exclusion. No language restrictions were imposed. Additional studies were also identified by reviewing the reference lists of the retrieved studies through a hand search.

Data on methodological issues, eligibility criteria, BCTs involved,^8^ interventions (including the number of participants treated, intervention provider) and study design, study duration, follow-up, comparisons, outcome measures, results, withdrawals, and adverse events were extracted independently by two review authors (Table 1).

**Table 1.** Information on included studies, risk of bias, and PEDro score

**Study**	**Sample (*n*)**	**Drop-outs**	**Population**	**Setting**	**Intervention design**	**Follow-up duration**	**Control**	**Main outcomes**	**PEDro score^a^**
Jolly *et al*^15^	525	Home programme: 6-month data, *n*= 246 (11 DNA, three died, three withdrew). At 12 months, *n*= 239 (14 DNA, four withdrew). Hospital programme: 6-month data, *n*= 239 (18 DNA, two died, three withdrew). At 12 months, *n*= 236 (20 DNA, one died)	After acute MI, coronary revascularisation or CABG	UK	HM for patients covering risk factor management. Telephone follow-up	12 months	Hospital-based CR	Home-based CR comparable to hospital-based CR in CVD risk factor improvements at 12 months of follow-up. Similar costs in running each programme	9
Dalal *et al*^16^	230: 104 into randomised arm, and 126 to preference arm	9-month follow-up data were available for 84/104 (81%) randomised, and 100/126 (79%) preference patients	Hospitalised for acute MI	UK	HM for 6 weeks. Cardiac rehab nurse made one home visit in first week after discharge, followed up by telephone calls over 6 weeks (typically one call in weeks 2, 3, 4, and 6)	9 months	Hospital-based CR	Home-based CR (HM) as effective as hospital-based CR in improving modifiable CVD risk factors	8
Zutz *et al*^19^	15: seven for usual care, and eight for home-based intervention	Two drop-outs by the end of the study for the usual care group	On a waiting list for cardiac rehabilitation, living within 60 km of site	Canada	Internet-based intervention with education modules, email communication with case manager and, dietician optional online discussion group, and entry of health behaviour data to monitor self-progress	12 weeks	No active treatment	The home-based CR programme group significantly improved modifiable CV risk factors compared to controls	8
Sinclair *et al*^23^	324: 163 in intervention and 161 in control groups	134/163 (82%) in intervention group. 133/161 (82.6%) in the control group	Discharged from hospital with acute MI, and ≥65 years old	UK	At least two home visits from trained support staff nurse to encourage patients around compliance, risk factor reduction, advice on stress, exercise, smoking cessation, and diet. Visits supplemented by telephone support and manual	100 days	Hospital-based cardiac rehabilitation	Significant improvement in confidence and self-esteem in the home-based group, although comparable improvements in CVD risk factors between home-based and centre-based CR	8
Lie *et al*^24^	203	93/101 (92%) in intervention group. 92/102 (90%) in control group	Patients with ischaemic heart disease (post-CABG patients)	Norway	A psychoeducative intervention, consisting of structured information and psychological support. All patients in the intervention group received two 1 hour home visits at 2 and 4 weeks after surgery	6 months	Standard discharge care that involved a non-standardised talk with the nurse/doctor	Home-based CR comparable to control group in terms of improving quality of life and activities of daily living	8
Wang *et al*^14^	160	Intervention group: at 6 months, *n*= 68/80 (85%). Control group: at 6 months, *n*= 65/80 (81%)	Patients who are post-MI	China	A home-based cardiac rehabilitation programme using a self-help manual, the HM, developed by the researchers. Patients had a 1-hour introduction to the manual, and telephone follow-up at 3 weeks	6 months	Usual care, hospital- based cardiac rehabilitation	Home-based CR (HM) improves quality of life and reduces anxiety compared to usual care for patients who are post-MI	8
Lee *et al*^17^	81	No data on drop-outs	Patients who are post-MI or with coronary revascularisation	UK	The home-based programme is nurse facilitated (with home visits and telephone contact), using the HM	3 months	Hospital supervised exercise sessions twice weekly for 12 weeks	Home-and hospital-based CR showed comparable improvements in haemostatic indices and CVD risk factors	9
Piotrowicz *et al*^20^	152	75/77 (97%) for home-based intervention. 56/75 (75%) for the control	Patients with heart failure	Poland	Home-based telemonitored rehabilitation based on continuous walking training on level ground. Patients wore an ECHO3 device which allowed remote ECG recording of the participant by the researchers	8 weeks	Control group: standard interval training on a cycle ergometer. Both groups: trained three times a week. All patients and their partners participated in an education programme	Home-based CR equally as effective centre-based CR for patients with heart failure, although better adherence in home-based group	8
Oerkild *et al*^21^	75 patients	30/36 (83%) for home-based intervention. 34/39 87%) for the control	Patients ≥65 years old with ischaemic heart disease	Denmark	For home-based, programme a physio visited twice within a 6-week interval to develop a training programme that could be performed at home and in the surrounding outdoor area. All patients received counselling and medical adjustment from a cardiologist at baseline and after 3, 6, and 12 months	12 months	The centre-based CR consisted of a 6 week group-based supervised exercise training for 60 minutes, twice a week, and patients were also encouraged to exercise at home	Home-based CR as effective as centre-based CR in improving exercise capacity, CVD risk factors, and health-related quality of life	8
Varnfield *et al*^18^	120 patients	For intervention, *n*= 46/60 (77%). For control, *n*= 26/60 (43%)	Patients who are post-MI	Australia	CR delivered at home: health and exercise monitoring, motivational and educational materials. Weekly mentoring consultations for 6 weeks, via telephone (approx. 15 mins each)	6 months	Traditional hospital-based CR (TCR)-two supervised exercise and 1hour educational sessions weekly for 6 weeks at one of four community centres	Home-based CR had better uptake, adherence and completion rates than centre-based CR. Comparable improvements in CVD risk factors in both groups	8
Oerkild *et al*^22^	40 patients	19/19 (100%) for the home-based intervention. 17/21 (81%) for the control	≥65 years with coronary heart disease	Denmark	Physiotherapist in home visits developed individualised exercise programme for home and surrounding outdoor area. Risk factor intervention, medical, physical, and psychological adjustments at baseline, 3, 6, and 12 months	12 months of follow-up, and mortality data after 5.5 years	Usual care with no rehabilitation for those who declined participation in centre-based CR	Home-based CR programme group significantly improved 6MWT performance at 3 months compared to controls	8

a PEDrO score maximum = 11. CABG = coronary artery bypass graft. CVD = cardiovascular disease. CR = cardiac rehabilitation. DNA = did not attend. ECG = electrocardiogram. HM = Heart Manual. MI = myocardial infarction. PEDro = Physiotherapy Evidence Database. 6MWT = 6-minute walk test.

There was no blinding to study author, institution, or journal.

### Assessment of quality, and risk of bias

The Physiotherapy Evidence Database (PEDro) scale^12^ (Table 1) was used to assess the quality of studies included in the review. In addition, two authors independently assessed each study that was included for risk of bias (‘high’, ‘low’ or ‘uncertain’) using the risk of bias tool, following guidance from the *Cochrane Handbook of Systematic Reviews of Interventions*,^13^ with a third review author acting as arbitrator as required.

### Measures of treatment effect

For each study, relative risk and 95% confidence intervals (CIs) were calculated for dichotomous outcomes, and mean differences and 95% CIs were calculated for continuous outcomes. When continuous outcomes were pooled on different scales, standardised mean differences were used.

Where available, changes from baseline (mean change scores) were used in preference to follow-up scores. When combining results for the individual studies, the authors generally used mean differences and a random effects model due to the clinical variation within studies.

### Assessing for heterogeneity

Diversity across the studies was assessed qualitatively in terms of intervention (content, duration, frequency, provider, and setting), participant demographic characteristics, outcome measures, and follow-up.

If two or more studies were considered clinically homogenous according to the above terms, data were assessed for statistical heterogeneity using RevMan (version 5.1).

The χ^2^ test was used in conjunction with the I^2^ statistic, which describes the percentage of variability in effect estimates due to heterogeneity. The level of significance for the χ^2^ test was set at *P* < 0.1.

### Data synthesis

Careful consideration was given to the appropriateness of conducting a meta-analysis. Data were summarised statistically when the data were available, and were sufficiently similar and of sufficient quality, and the statistical analysis was performed in accordance with guidelines (version 5.1.0).^13^

### Behaviour change techniques (BCTs)

To allow a greater understanding of what behavioural techniques were used in this patient population, two trained review authors independently screened the articles included, and extracted BCTs using Michie’s BCT taxonomoy.^8^

## RESULTS

**Figure 1.**
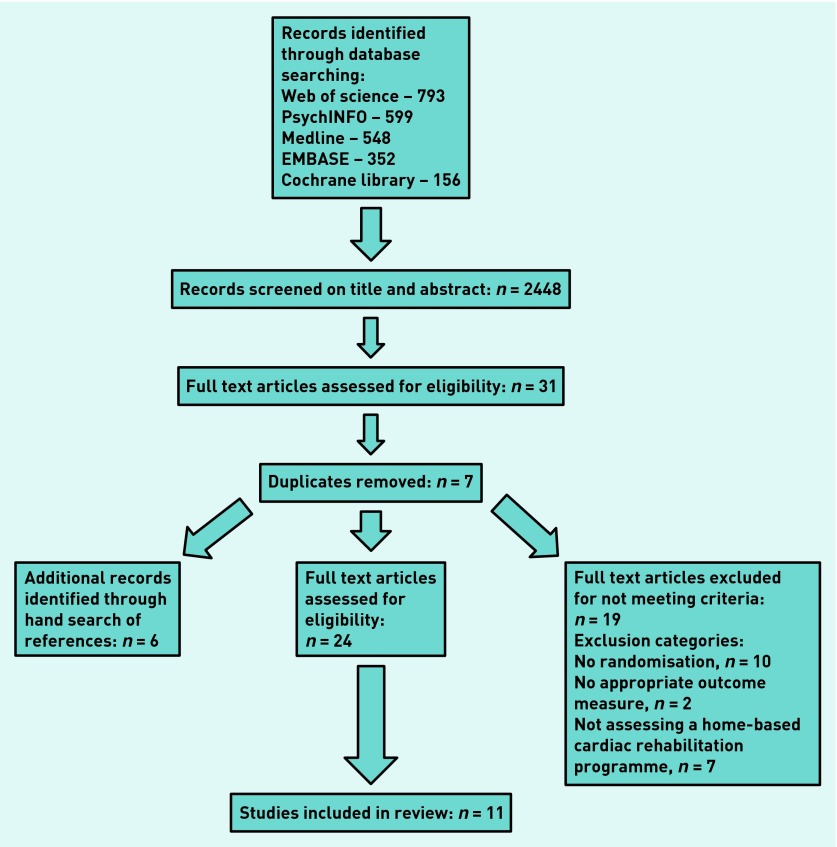
***Flow diagram of reviewed and included studies.***

The search criteria returned 2448 articles and the authors reviewed the full text articles of 31 studies, identifying 24 possible studies for inclusion in this review (Figure 1). From a hand search of the reference lists of the 24 studies, six additional potentially eligible studies were identified. In all, 11 studies were included in the review. Of the 19 excluded, 10 were excluded because there was no randomisation, seven were excluded because they did not assess a home-based CR programme, and two were excluded as there was no appropriate outcome measure.

### Programme design and evaluation

Of the 11 studies included, four used the Heart Manual as their home-based CR programme.^14^^–^17 Three used technology to assist delivery of the home-based CR intervention, including a smartphone,^18^ the internet,^19^ and telemonitoring.^20^ In the remaining four studies, CR was delivered in the participant’s home by physiotherapists^21^^,^^22^ or nurses.^23^^,^^24^

In seven studies the control group was hospital- or centre-based CR,^15^^–^18^,^^20^^,^^21^^,^^23^ while in two studies the control groups received ‘usual care’,^14^^,^^24^ and in two they received no active treatment^19^^,^ 22 (Table 1). One study was conducted in each of the following countries Canada,^19^ Australia,^18^ Poland,^20^ Norway,^24^ and China,^14^ two in Denmark,^21^^,^^22^ and four in England.^15^^–^17^,^^23^

In 10 studies, patients were post-MI or post-angioplasty/coronary artery bypass graft (CABG), and one study exclusively included patients with heart failure.^20^ All participants were recruited from secondary care. Outcomes were assessed from 8 weeks^20^ to 1 year.^15^^,^^21^^,^^22^

### Behavioural change techniques in the included studies

As there were only a limited number of studies with comparable outcome data, it was not possible to compare the relative effectiveness of different BCTs or to conduct a meta-regression of the BCTs.^13^ The BCT identified as social support (unspecified) was employed in all 11 studies, while the BCT goal setting (behaviour) was employed in 10 studies (Table 2). The Heart Manual intervention used the BCTs of goal setting (behaviour), monitoring of behaviour by others without feedback, self-monitoring of behaviour, self-monitoring of outcome(s) of behaviour, social support (unspecified), instruction on how to perform the behaviour, pharmacological support, and reducing negative emotions. All studies except one — Lie and colleagues^24^ — reported significant positive effects in change of measured CVD risk factors, and the BCTs used in the successful programmes generally included instructions on how to perform the behaviour, BCTs related to monitoring, and credible source. The BCTs employed in Lie and colleagues^24^ were social support, goal setting, reducing negative emotions, pharmacological support, information about health, and problem solving.

**Table 2.** Behaviour change techniques (BCTs) used by the studies included in the review

**BCT label^8^**	**BCT group**	**Example of how the BCT was used**	**Frequency of use**	**Studies where found**
3.1 Social support (unspecified)	Social support	*‘... motivational and educational materials to participants via text messages ...’* 20	11	16^–^24^,^^25^^,^^26^
1.1 Goal setting (behaviour)	Goals and planning	*‘Exercise targets were at least 30 min of moderate activity...’* 20	10	16^–^20^,^^22^^–^25
11.2 Reduce negative emotions	Regulation	*‘… relaxation and stress management techniques ...’* 19	7	16^–^20^,^^25^^,^^26^
4.1 Instruction on how to perform the behaviour	Shaping knowledge	‘Patients were carefully instructed in the training programme ...’ 24	7	16^–^19^,^^23^^–^25
2.1 Monitoring of behaviour by others without feedback	Feedback and monitoring	*‘... used a smartphone for health and exercise monitoring ...’* 20	6	16^–^20^,^^23^
2.3 Self-monitoring of behaviour	Feedback and monitoring	*‘Participants were asked to wear their heart rate monitors ... and upload their exercise data at least twice per week onto the website.’* 21	6	16^–^21
2.4 Self-monitoring of outcome(s) of behaviour	Feedback and monitoring	*‘Each participant was equipped with a smartphone ... with health diary and activity monitoring applications, blood pressure monitor and weight scale.’* 20	6	16^–^21
9.1 Credible source	Comparison of outcomes	*‘... monthly ask-an-expert group chat sessions.’* 21	6	18^,^^21^^–^25
11.1 Pharmacological support	Regulation	*‘… nurse counselled patients ..., giving information on … drug treatment.’* 18	6	16^–^19^,^^23^^,^^26^
5.1 Information about health consequences	Natural consequences	*‘… simple explanations about coronary heart disease …’* 18	5	18^–^20^,^^25^^,^^26^
2.5 Monitoring of outcome(s) of behaviour without feedback	Feedback and monitoring	*‘Regarding risk factor intervention and medical adjustment, the patients consulted a cardiologist at baseline and after 3, 6, and 12 months.’* 24	3	21^,^^22^^,^^24^
3.2 Social support (practical)	Social support	*‘… technical phone support during the trial if required.’* 20	3	19^,^^20^^,^^25^
12.5 Adding objects to the environment	Antecedents	*‘Each participant was equipped with a smartphone …’* 20	3	18^–^20
1.2 Problem solving	Goals and planning	*‘… self-help treatments for psychological problems commonly experienced by patients with myocardial infarction …’* 19	2	19^,^^26^
2.2 Feedback on behaviour	Feedback and monitoring	*‘Mentors reviewed participants’ updated data prior to weekly consultations ... to provide informed, personalised feedback on progress ...’* 20	2	20^,^^22^
2.6 Biofeedback	Feedback and monitoring	*‘Before beginning a training session, patients … used the mobile phone to answer a series of questions regarding their present condition, including fatigue, dyspnoea, blood pressure, body mass, and medication taken. Patients then transmitted resting ECG data to the monitoring centre. If no contraindications to training were identified, patients were given permission to start the training session.’* 22	2	20^,^^22^
5.6 Information about emotional consequences	Natural consequences	*‘... specific self-help treatments for psychological problems commonly experienced by patients with myocardial infarction...’* 19	2	18^,^^19^
6.1 Demonstration of the behaviour	Comparison of behaviour	*‘A physiotherapist made home visits … in order to develop a training programme that could be performed at home and in the surrounding outdoor area.’* 23	2	23^,^^25^
8.1 Behavioural practice/rehearsal	Repetition and substitution	*‘In order to prescribe adequate exercise programmes, a 6-minute walk test and a maximal symptom-limited exercise capacity test on bicycle ergometer … was conducted. The main types of stationary exercise recommended were self-paced brisk walking and cycling.’* 23	2	23^,^^25^
2.7 Feedback on outcome(s) of behaviour	Feedback and monitoring	*‘… personalised feedback on progress according to goals set.’* 20	1	20

Within the BCT taxonomy,^8^ individual BCTs are clustered into hierarchical groups that commonly appear together in behavioural interventions. The commonest group of BCTs used in the 11 included studies was feedback and monitoring, while the second most common group was social support. Of all the groups of BCTs listed in the taxonomy,^8^ six were not identified as having been used within any of the studies. These were associations, reward and threat, identity, scheduled consequences, self-belief, and covert learning.

### Quality and risk of bias

Using the PEDro scale^12^ (Table 1), all studies included were deemed to be of high methodological quality. All were randomised controlled trials with a low risk of bias.^13^ The study by Dalal and colleagues^16^ also had a patient-preference arm so that only data for the randomised patients were included in the meta-analysis. Jolly and colleagues^15^ and Lee and colleagues^17^ were the only two studies in which assessors were blinded to the outcome measures. Study follow-up overall varied from 77%^18^ to 100%,^17^^,^^22^ and all studies fully accounted for the study participants, and provided reasons for any missing data. The authors identified no other potential sources of bias.

### Assessment of reporting bias

**Figure 2.**
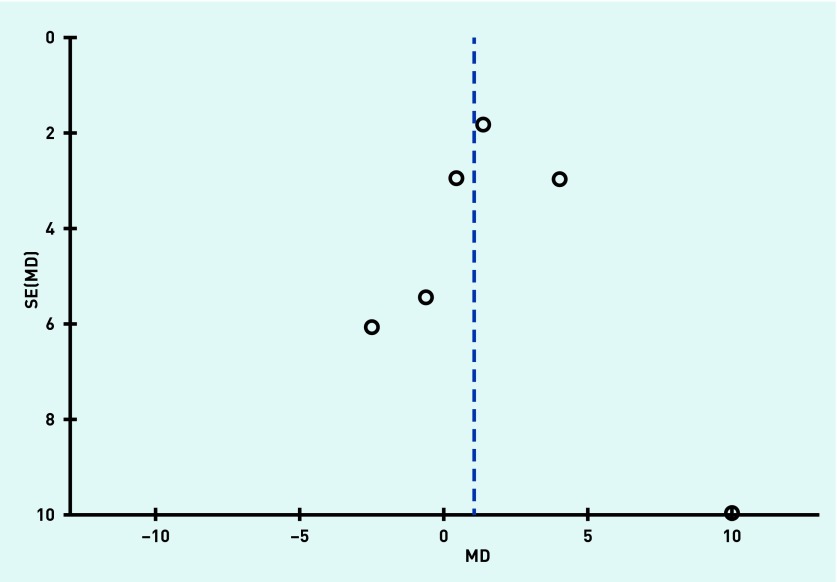
***Funnel plot to assess reporting bias for the variable systolic blood pressure. MD = mean difference. SE = standard error.***

**Figure 3.**
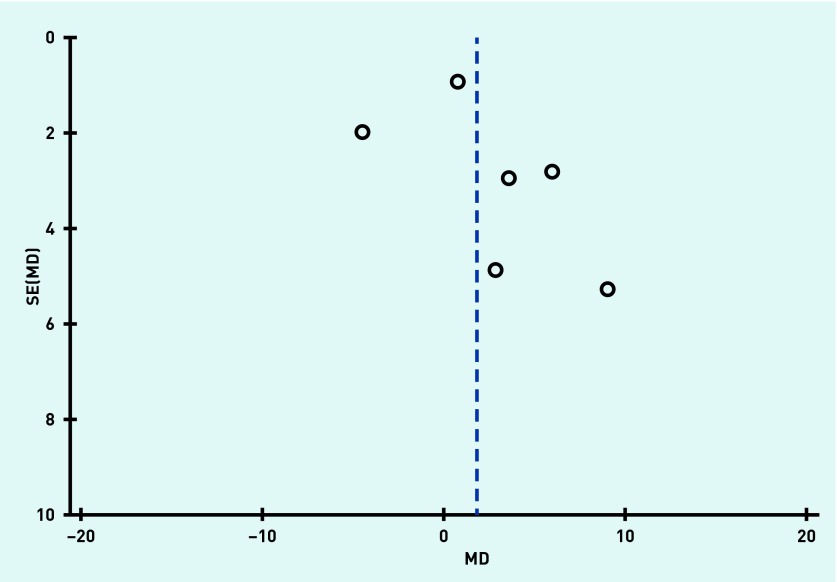
***Funnel plot to assess reporting bias for the variable diastolic blood pressure. MD = mean difference. SE = standard error.***

Funnel plots were produced to assess reporting bias and no obvious reporting bias was found, as illustrated in Figure 2 and Figure 3 in respect of systolic blood pressure and diastolic blood pressure outcomes.

### Effects of interventions

All but one study^24^ reported the positive effect of home-based CR on modifiable CVD risk factors. For comparison of the effectiveness of home-based and comparator programmes the authors combined outcome data from all types of comparator groups in the included studies. For both studies by Oerkild and colleagues^21^^,^^22^ the data at the 3-month review — which reported the change from baseline — were used for meta-analysis, and in the study by Dalal and colleagues^16^ only randomised data were used.

**Figure 4.**
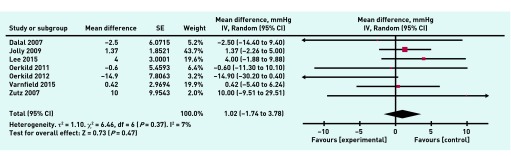
***Meta-analysis of resting systolic blood pressure. df = degrees of freedom. IV = inverse variance of the treatment effect. Random = random effects model. SE = standard error of the treatment effect.***

**Figure 5.**
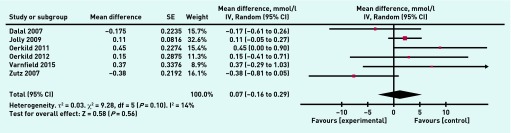
***Meta-analysis of total cholesterol. df = degrees of freedom. IV = inverse variance of the treatment effect. Random = random effects model. SE = standard error of the treatment effect.***

**Figure 6.**
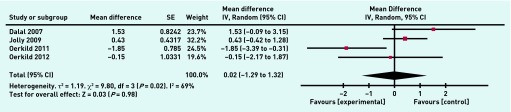
***Meta-analysis for the anxiety section of the HADS questionnaire. df = degrees of freedom.*** ***IV = inverse variance of the treatment effect. Random = random effects model. SE = standard error of the treatment effect.***

A meta-analysis was undertaken on eight individual variables to compare outcomes between home-based and comparator programmes in the included studies. There was no significant difference between home and hospital/centre-based CR in their effects on resting systolic blood pressure (1.02 mmHg, 95% CI = 1.74 to 3.78, *P* = 0.3) (Figure 4), resting diastolic blood pressure (−0.89 mmHg, 95% CI = 4.35 to 2.58, *P* = 0.62), peak VO_2max_ (greatest volume of oxygen that the body can consume per unit time) (1.19 ml/kg/min, 95% CI = 0.78 to 3.16, *P* = 0.24) and the distance covered in the 6-minute walk test (8.47 m, 95% CI = 10.98 to 27.92, *P* = 0.39). There was also no significant difference between home and hospital/centre-based CR in terms of overall treatment effect for total cholesterol (0.07 mmol/l, 95% CI = −0.16 to 0.29, *P* = 0.56) (Figure 5), HDL-cholesterol (0.01 mmol/l, 95% CI = 0.06 to 0.07, *P* = 0.79), and LDL-cholesterol (0.02 mmol/l, 95% CI = 0.25 to 0.29, *P* = 0.88), or for the anxiety (0.02, 95% CI = −1.29 to 1.32, *P* = 0.98) (Figure 6) and the depression sections of the HADS (hospital anxiety and depression score) questionnaire (−0.21, 95% CI = −0.74 to 0.32, *P* = 0.44).

## DISCUSSION

### Summary

This systematic review comprised 11 randomised controlled studies reviewing the use of home-based CR programmes for patients with CVD and the comparator programmes, the majority of which were centre- or hospital-based CR. This is the first review to collate observations on the use of BCTs within home-based CR programmes, including the Heart Manual. A total of 20 different BCTs were used in the 11 included studies, with the BCT identified as social support (unspecified) being used in all 11 studies, while the BCT goal setting (behaviour) was employed in all but one of the included studies. The BCT profile related to monitoring, instruction on how to perform the behaviour, and credible source were generally included in effective programmes. The Heart Manual intervention used the BCTs of goal setting (behaviour), monitoring of behaviour by others without feedback, self-monitoring of behaviour, self-monitoring of outcome(s) of behaviour, social support (unspecified), instruction on how to perform the behaviour, pharmacological support, and reduce negative emotions, and was consistently effective in modifying CVD risk factors in the included studies.

This review offers new information about the use of BCTs within home-based CR programmes for patients with CVD, illustrating frequent use of the BCTs of social support (unspecified) and goal setting (behaviour) within programmes that have been shown to be effective in reducing CVD risk factors. However, further robust trials that describe and evaluate the use of BCTs,^8^ building on NICE guidance,^7^ are required in order to refine the design of home-based CR programmes for optimal secondary prevention of CVD.

### Strengths and limitations

The authors have attempted to identify all studies of potential relevance to this review by developing a comprehensive search strategy and then supporting this through hand searching reference lists of all the full text articles included and excluded from the review. Visual inspection of funnel plots provided little evidence for publication bias. The authors sought to include all eligible studies regardless of publication language, although all studies included were in English.

A limitation is that studies included in this review lacked consistency in the outcome measures used, and in duration of follow-up. Combining all results in a meta-analysis was therefore difficult. This review generally pooled data collected at the end of the study per protocol. However, follow-up duration varied from 8 weeks to 1 year. The results should therefore be interpreted with caution, as shorter study durations may not allow sufficient time for the rehabilitation interventions to produce an impact on modifiable vascular risk factors and early changes may not be sustained. Intervention intensity is generally a poorly-defined concept, as discussed in a previous Cochrane systematic review,^25^ and differences in intervention intensity are considered to be a source of heterogeneity within complex interventions.^25^ Intervention intensity was different across the included studies and could be a potential source of bias within the review. The studies were generally carried out in developed countries, so that the findings may not be applicable to developing countries.

### Comparison with existing literature

Previous studies have highlighted the comparable effects of home-based CR and hospital-based programmes.^2^^,^^26^^,^^27^ This study supports these findings by illustrating in the meta-analysis that home-based CR provides comparable improvements in CVD risk factors to other treatment options, including hospital-based approaches. Indeed all but one study illustrated the positive effect of home-based CR on modifiable CVD risk factors. However this study further develops these findings by including a wide range of patients with CVD including patients with heart failure and post-coronary vascularisation and by reviewing home-based CR programmes delivered using the latest technology (for example, smartphones), not previously reviewed.^2^ The findings are more up-to-date than previous reviews,^2^^,^^26^^,^^27^ being from 2005–2015, and therefore applicable to current medical treatments. Home-based cardiac rehabilitation programmes are generally attractive to patients due to their accessibility,^2^^,^^27^ helping to improve compliance.^28^ They also tend to be less costly than hospital- or centre-based programmes,^28^ and fit with the aim of shifting patient management from a hospital base into the community: a current theme within modern health care.

NICE published guidance in 2014 on individual-level behaviour change interventions for promoting change in modifiable cardiovascular risk factors in the public.^7^ These guidelines recommend that behaviour change programmes, including lifestyle management programmes, should include support for individuals to make change through the use of self-monitoring, goal setting, social support, and relapse prevention strategies, and the provision of relevant information on the health consequences of the behaviour. In keeping with these guidelines, the BCT social support (unspecified) was employed in all studies included in this review and the BCT goal setting (behaviour) was used in all but one.^19^ Few studies used self-monitoring and relapse prevention strategies. Indeed only six studies^14^^–^19 utilised the BCTs of self-monitoring of behaviour and self-monitoring of outcome(s) of behaviour. Of note, these BCTs were found to be used simultaneously. There is no specific BCT for relapse prevention, although this could be covered by multiple BCTs such as action planning.

### Implications for research and practice

Future studies aimed at developing home-based cardiac rehabilitation programmes for patients with CVD should therefore consider the techniques of self-monitoring, goal setting, social support, and relapse prevention strategies, recommended by NICE, to maximise the likelihood of establishing and maintaining new behaviours to optimise secondary CVD prevention. In particular, self-monitoring and relapse prevention strategies should be further developed and their impact on tackling modifiable CVD risk factors assessed.
